# Culturally Sensitive Hypertension Interventions: A Ghana Case Study

**DOI:** 10.1002/hsr2.70886

**Published:** 2025-06-11

**Authors:** John Patrick C. Toledo

**Affiliations:** ^1^ Department of Theology and Religious Education De La Salle University Manila Philippines

Dear Editor,

I've read with great interest the study entitled Interpersonal Factors Influencing Hypertension Control: A Cross‐Sectional Study Among Hypertensive Patients in the Ashanti Region, Ghana,^1^ which explores the interpersonal factors that hinder hypertension (HPT) management among patients in the Ashanti Region of Ghana. The study, conducted at the Komfo Anokye Teaching Hospital (KATH), involved 350 hypertensive patients and used a pretested questionnaire to collect data. In addition to highlighting important issues such as medication nonadherence, a lack of information about hypertension, and physical inactivity, the findings also point to education level, self‐efficacy, income, and marital status as important variables that affect how well HPT is managed.

According to the study,^1^ there is a high proportion of physical inactivity (66.6%), moderate understanding of hypertension (49.7%), and medication nonadherence (56.3%) among the participants. Higher self‐efficacy and tertiary education improve the ability to control hypertension, but higher perceived hurdles, divorce or separation, a high family income, a longer duration of the disease, and good knowledge of hypertension reduce the likelihood of successful treatment. The Ashanti Region's management of hypertension is greatly impacted by these interpersonal factors, highlighting the necessity of focused health education and self‐management programs.

Ghana and other low‐ and middle‐income countries (LMICs) are disproportionately affected by hypertension, a global public health concern. The results of the study are especially pertinent to low‐resource nations, where access to healthy lifestyles, healthcare, and education is frequently restricted. Because of things like poverty, lack of access to information, and inadequate healthcare infrastructure, the issues mentioned—including pharmaceutical nonadherence and inadequate knowledge—are prevalent in developing nations. Interventions can be customized to meet the unique requirements of these communities by taking into account these barriers.

The study offers a number of viable ways to raise the quality of life and better control hypertension for people in the Ashanti Region and comparable environments. Programs for targeted health education can encourage better lifestyles and increase drug adherence. Better condition management may also result from empowering and supporting patients to increase their sense of self‐efficacy. It is essential that intervention efforts take sociocultural aspects like marital status and ethnicity into account. Policies should also emphasize follow‐up treatment and incorporating education into interventions, particularly for individuals with prolonged disease durations. Healthcare professionals should make use of current coping strategies to enhance drug adherence, and community‐based awareness initiatives can increase general knowledge about hypertension.

In conclusion, the study emphasizes the urgent need for targeted, culturally sensitive interventions to improve hypertension management in the Ashanti Region of Ghana, with broader implications for similar low‐resource settings worldwide. Improving the quality of life for people with hypertension requires addressing interpersonal variables such as drug nonadherence, a lack of understanding, and physical inactivity. Qualitative research on the lived experiences of hypertensive patients should be incorporated into future studies to better understand the obstacles they encounter and create patient‐centered, more efficient methods of controlling their condition, which will ultimately improve everyone's quality of life.

## Author Contributions

**John Patrick C. Toledo:** conceptualization, writing – original draft, writing – review and editing.

## Ethics Statement

Ethical standards are followed in the research.

## Conflicts of Interest

The author declares no conflicts of interest.

## Transparency Statement

The lead author John P. C. Toledo affirms that this manuscript is an honest, accurate, and transparent account of the study being reported; that no important aspects of the study have been omitted; and that any discrepancies from the study as planned (and, if relevant, registered) have been explained.

## Data Availability

Data sharing is not applicable to this article as no new data were created or analyzed in this study.
